# Depression, anxiety, stress, and physical activity of Australian adults during COVID-19: A combined longitudinal and repeated cross-sectional study

**DOI:** 10.3389/fpsyg.2022.962962

**Published:** 2022-10-05

**Authors:** Wei Zhao, Corneel Vandelanotte, Saman Khalesi, Stephanie J. Alley, Sue L. Williams, Tanya L. Thwaite, Andrew S. Fenning, Robert Stanton, Quyen G. To

**Affiliations:** ^1^Physical Activity Research Group, School for Health Medical and Applied Science, Appleton Institute, Central Queensland University, Rockhampton, QLD, Australia; ^2^Cluster for Resilience and Wellbeing, School for Health Medical and Applied Science, Appleton Institute, Central Queensland University, Rockhampton, QLD, Australia

**Keywords:** mental health, DASS-21, exercise, COVID-19 pandemic, depressive symptoms, anxiety, distress

## Abstract

**Background:**

The COVID-19 pandemic has led to a worsening of mental health and health behaviors. While physical activity is positively associated mental health, there is limited understanding of how mental health and physical activity evolve throughout the COVID-19 pandemic. This study aimed to examine changes in depression, anxiety and stress and physical activity, and associations between depression, anxiety, and stress with physical activity in Australian adults across three-time points during the COVID-19 pandemic.

**Materials and methods:**

This study collected both longitudinal and cross-sectional data at three-time points during the COVID-19 pandemic in Australia (i.e., April, July/August, and December 2020). Australians aged 18 years and over were invited to complete online surveys hosted on Qualtrics survey platform. Linear mixed models with random subject effect and general linear models were used to analyze the longitudinal and repeated cross-sectional data respectively.

**Results:**

The number of participants in cross-sectional surveys and longitudinal surveys was 1,877 and 849, respectively. There was an overall reduction between time 2 vs. time 3 in depression (*d* = 1.03, 95% CI = 0.20, 1.85), anxiety (*d* = 0.57, 95% CI = 0.02, 1.12), and stress (*d* = 1.13, 95% CI = 0.21, 2.04) scores but no significant differences in physical activity across three-time points. On average, participants who met the physical activity guidelines had lower depression (*d* = −2.08, 95% CI = −2.90, −1.26), anxiety (*d* = −0.88, 95% CI = −1.41, −0.34), and stress (*d* = −1.35, 95% CI = −2.13, −0.56) scores compared to those not meeting the guidelines.

**Conclusion:**

In the context of the ongoing COVID-19 pandemic, both governments and service providers should continue to provide the public with timely mental health support and promote the benefits of physical activity, as a cost-effective strategy to improve mental health and wellbeing.

## Introduction

On March 11, 2020, the World Health Organization (WHO) declared the novel coronavirus (COVID-19) a pandemic, due to its rapid global spread and high mortality (World Health Organisation, 2021). Since then, restrictive measures to contain the virus including lockdowns, self-isolation, and quarantine have brought significant social and economic impact to people’s lives (Bonaccorsi et al., 2020; Dong and Bouey, 2020; Wells et al., 2020). As a consequence of these measures, mental health outcomes and health behaviors have declined (Xiong et al., 2020).

Studies exploring the impact of the COVID-19 pandemic on mental health have reported an increased prevalence of mental health problems and psychological distress in both clinical and general populations across the world, including depression, anxiety, stress, and post-traumatic disorder (Ahmed et al., 2020; Brooks et al., 2020; Chandola et al., 2020; Salari et al., 2020; Serafini et al., 2020; Torales et al., 2020; Violant-Holz et al., 2020; Xiong et al., 2020; Passavanti et al., 2021). Systematic reviews and meta-analyses on the impact of COVID-19 on mental health in the general population have reported a two-to-eight-fold increase in the prevalence of depression, anxiety, stress, and sleep disorders (Luo et al., 2020; Salari et al., 2020; Nochaiwong et al., 2021; Wu et al., 2021). This is concerning given that mental health is integral for good physical health, psychosocial functioning, and quality of life (World health Organisation, 2018).

Longitudinal studies were also examined changes in mental health over time during the pandemic. Specifically, a UK longitudinal study reported an almost threefold increase in mental health problems in the general population between the pre-pandemic period (2017–2019) and April 2020, with only a slight reduction in the elevated prevalence rate and severity by May/June 2020 (Daly et al., 2020). Similarly, an Austrian longitudinal study reported elevated levels of depression, anxiety and stress found in the general population in April 2020, largely unchanged by September 2020 (Pieh et al., 2021). In contrast, a USA longitudinal study found a significant increase in psychological distress in the general population at the beginning of the pandemic (March/April 2020), but by June 2020 psychological distress had reduced to be close to pre-pandemic levels (Daly and Robinson, 2021). As changes in mental health are generally attributed to governments’ pandemic response policies, population resilience, and infection and mortality rates which were different worldwide (Daly et al., 2020; Daly and Robinson, 2021; Pieh et al., 2021), it is important to have local data to better understand the pandemic impact on mental health in Australia. To our knowledge there are no longitudinal studies examining change in mental health during the pandemic in Australia.

Previous studies have also reported significant declines in physical activity following implementation of lockdown measures, as people have been unable to access recreational and gym facilities and engage in incidental physical activities (Violant-Holz et al., 2020; Faulkner et al., 2021; Karageorghis et al., 2021). One of the few longitudinal studies in this area tracked a sample of 35,915 adults in England from March 2020 to August 2020 and reported that the majority of participants either stayed physically inactive or reduced physical activity from pre-lockdown to lockdown, and then to post lockdown (Bu et al., 2021). An Australian longitudinal study also reported a significant reduction albeit small in steps logged by participants of the Australian 10,000 steps program after the national lockdown in April 2020, but with a gradual recovery leading up to June 2020, as social restrictions eased (To et al., 2021).

There is evidence demonstrating that an increase of physical activity is associated with lower levels of depression, anxiety, and stress (Rebar et al., 2015; Chekroud et al., 2018; Schuch et al., 2019; Jacob et al., 2020; Kandola et al., 2020; Stanton et al., 2020; Violant-Holz et al., 2020; Puccinelli et al., 2021). However, the mechanism for mental health benefits of physical activity is not well-established. The effects are likely resulted from a combination of several mechanisms at psychological (e.g., mood, feelings of mastery, self-efficacy) and neurophysiological (e.g., hippocampal neurogenesis, hypothalamic-pituitary adrenal axis regulation) levels (Rebar et al., 2015). As the extraordinary circumstances caused by COVID-19 had a profound impact on people’s physical and mental health, it is possible that the mechanism that normally drive the association between physical activity and mental health were altered. To date, there have been fewer studies examining the association between changes in physical activity with depression, anxiety, and stress during the COVID-19 pandemic, hence more work is needed in this space. For example, a Canadian study found that those who reported engaging in more physical activity during lockdown periods experienced lower anxiety than those who reported being less physically active during lockdown (Lesser and Nienhuis, 2020).

Given the ongoing threats of the pandemic and the importance of mental health and physical activity for our overall health, the present study aimed to examine changes in depression, anxiety and stress, and physical activity, as well as the associations between depression, anxiety and stress with physical activity in Australian adults across three-time points throughout the pandemic. The findings from this study can assist governments and service providers making the decision regarding providing mental health and physical activity support for the public during the COVID-19 pandemic.

## Materials and methods

### Study design

This study made use of both longitudinal (i.e., the same participants completing at least two surveys) and repeated cross-sectional (i.e., different participants completing the survey at different time points) data collected at three-time points during COVID-19 pandemic in Australia. During the first survey, which was conducted from 9th to 19th April 2020, Australian governments had implemented nationwide lockdown measures to reduce the spread of infection including social distancing, stay at home orders, and domestic and international incoming and outgoing travel restrictions. During the second survey, which was conducted from 30th July to 16th August 2020, Victoria was the only Australian state in which a second wave of the COVID-19 pandemic had occurred, and a Victorian state-wide lockdown measure was enforced to protect people and control the rate of infection. In contrast, all other Australian states had reduced their preventive measures due to the low number of infected cases. During the third survey (conducted from 1st to 25th of December 2020), there were no hard lockdowns in place anywhere in Australia.

### Participants and recruitment methods

Participants were recruited *via* paid advertisements on Facebook and through social media (the account was “CQUni COVID-19 Community Study” which is not connected to the official CQUni page). Advertisements included a direct link to the surveys hosted on Qualtrics survey platform. A range of images to separately target males and females were used. Central Queensland University internal emails including academic and professional staff were also used for recruitment. Australians aged 18 years and over were invited to complete the surveys. Online informed consent was obtained from all participants after they were provided with the information on the nature of the study and participation. The survey responses were anonymous. Participants were not paid for participation and no information about whether participants were participating in other studies was collected. Participants who completed the first or second survey were invited to participate in subsequent surveys. Those consenting to being re-contacted, provided their e-mail address so they could be advised of subsequent survey rounds. The total number of participants in three cross-sectional surveys were 1887 and in longitudinal surveys (including new participants recruited in survey 2) were 849. The study was approved by the Human Research Ethics Committee of Central Queensland University, Australia (Approval number 22332).

### Measures

Depression, anxiety, and stress were assessed using the 21-item Depression, Anxiety and Stress Scale (DASS-21) (Lovibond and Lovibond, 1995). The DASS-21 has good construct validity in measuring the dimensions of depression, anxiety, and stress, as well as a high reliability with Cronbach’s alpha α of 0.88, 0.82, and 0.90 for depression, anxiety, and stress respectively for general adult population (Henry and Crawford, 2005). Each scale comprises seven items scored on a 4-point Likert scale ranging from 0 (did not apply to me at all) to 3 (applied to me very much, or most of the time). The score for each scale was calculated by adding the scores of the relevant seven items and multiplying the total by two. For clinical purpose, the following cut-off scores have been developed for defining (1) normal, (2) mild, (3) moderate, (4) severe, and (5) extremely severe scores respectively for each DASS scale: depression (0–9, 10–13, 14–20, 21–27, 28+), anxiety (0–7, 8–9, 10–14, 15–19, 20+), and stress (0–14, 15–18, 19–25, 26–33, 34+).

Physical activity was measured using the 8-item Active Australia Survey (AAS) (Australian Institute of Health and Welfare, 2003), which asks participants to report frequency and duration of their physical activity over the past 7 days, including gardening, walking, moderate, and vigorous physical activities. The total time in physical activity was calculated by adding the minutes for walking and moderate activity and double the time spent in vigorous activity (not including gardening). The AAS has an acceptable criterion validity with correlations between self-report physical activity and accelerometer data and pedometer steps 0.52 and 0.43, respectively (Brown et al., 2004). Physical activity level was coded to: meet (total physical activity ≥ 150 min per week); or not meet (total physical activity < 150 min per week) the national physical activity guidelines (Australian Institute of Health and Welfare, 2003).

Sociodemographic characteristics included in this study were age, gender, educational attainment, household income, Body Mass Index (BMI), and chronic disease status. Participants’ weekly household income was categorized as: (1) less than AUD $1,000; (2) between AUD $1,000 and 2,000; (3) more than AUD $2,000. BMI was calculated as weight in kilograms divided by height in meters squared and interpreted based on the standard weight status categories: Normal (24.9 kg/m^2^ or lower), Overweight (BMI of 25.0–29.9 kg/m^2^), and Obese (BMI of 30.0 kg/m^2^and above). When assessing for the presence of chronic disease, participants were asked the question “Have you ever been told by a doctor that you have any chronic health problems?” with a “yes” or “no” response.

### Statistical analysis

Statistical analysis was conducted using Statistical Package for the Social Sciences (SPSS) version 27. Descriptive statistics (mean ± standard deviation [M ± SD] and percentages [n, %]) were used to describe mental health and physical activity status, and sociodemographic characteristics of the sample at each of the three-time points.

To examine changes in depression, anxiety, stress and physical activity, and associations between depression, anxiety, and stress with physical activity across three-time points, linear mixed models with random subject effect were employed for the longitudinal data and general linear models were employed for the repeated cross-sectional data. Robust variance estimator was also used.

Sociodemographic characteristics were accounted for in multivariable analysis. There were less than 1% missing values for sociodemographic characteristics factors, except for household income which was 16.9% for repeated cross-sectional data, and 12.9% for longitudinal data across three-time points. Therefore, two multivariable models were run with and without household income. As the result between the two models were similar, only the multivariable model with household income is presented here. Given there were three-time points, Bonferroni adjustment method was used to correct inflated *p*-values. Crude estimates and those adjusted for potential confounders were reported with 95% confidence interval. Significance level was set at 0.05 and all *p*-values were two-tailed.

## Results

### Participants characteristics

Sociodemographic characteristics of the longitudinal sample are shown in Table 1. At baseline, two-thirds of participants (68.7%) were female and reported being overweight or obese (67.6%). Close to half of the participants reported a weekly household income of more than $2,000 Australian dollars per week and having at least one chronic condition. Average age was 52.3 years (SD = 14.2). For all time points, most participants were in the normal to mild ranges for depression, anxiety, and stress and close to half of the participants were meeting the physical activity guidelines. The characteristics of the repeated cross-sectional samples were similar to the longitudinal (Table 2).

**TABLE 1:** Sociodemographic characteristics of the longitudinal sample.

	Survey 1		Survey 2		Survey 3	
	n	% or mean (SD)	n	% or mean (SD)	n	% or mean (SD)
**Gender**						
Male	199	31.3%	270	31.8%	162	29.6%
Female	436	68.7%	579	68.2%	386	70.4%
Age (years)	635	52.29 (14.18)	849	53.22 (14.13)	548	53.81 (13.88)
Years of education	635	16.53 (4.68)	849	16.62 (4.69)	548	16.52 (4.62)
**Household income/week**						
<1,000AUD	147	26.5%	212	28.7%	141	29.7%
1,000–<2,000AUD	175	31.5%	217	29.4%	136	28.6%
≥2,000AUD	233	42.0%	310	41.9%	198	41.7%
**Chronic disease**						
Yes	300	47.2%	418	49.2%	281	51.3%
No	335	52.8%	431	50.8%	267	48.7%
**Weight status**						
Normal	197	31.2%	262	31.0%	149	27.3%
Overweight	214	33.9%	275	32.5%	198	36.3%
Obese	213	33.7%	293	34.7%	186	34.1%
**Depression level**						
Normal	408	64.3%	539	63.5%	368	67.2%
Mild	68	10.7%	82	9.7%	66	12.0%
Moderate	78	12.3%	109	12.8%	49	8.9%
Severe	34	5.4%	50	5.9%	23	4.2%
Extremely severe	47	7.4%	69	8.1%	42	7.7%
**Anxiety level**						
Normal	514	80.9%	652	76.8%	434	79.2%
Mild	29	4.6%	54	6.4%	27	4.9%
Moderate	50	7.9%	71	8.4%	58	10.6%
Severe	14	2.2%	30	3.5%	13	2.4%
Extremely severe	28	4.4%	42	4.9%	16	2.9%
**Stress level**						
Normal	464	73.1%	642	75.6%	432	78.8%
Mild	65	10.2%	63	7.4%	35	6.4%
Moderate	46	7.2%	62	7.3%	36	6.6%
Severe	45	7.1%	49	5.8%	29	5.3%
Extremely severe	15	2.4%	33	3.9%	16	2.9%
**Meeting physical activity guidelines**						
No	276	43.5%	376	44.9%	226	41.7%
Yes	359	56.5%	461	55.1%	316	58.3%
Depression score	635	8.68 (9.82)	849	8.98 (10.44)	548	7.93 (10.31)
Anxiety score	635	4.10 (6.27)	849	4.60 (6.76)	548	4.00 (5.96)
Stress score	635	9.88 (9.45)	849	9.92 (9.89)	548	8.89 (9.52)
Total physical activity minutes/week	635	322.60 (366.56)	837	312.89 (361.53)	542	330.35 (359.94)

**TABLE 2:** Sociodemographic characteristics of the cross-sectional sample.

	Survey 1		Survey 2		Survey 3	
	n	% or mean (SD)	n	% or mean (SD)	n	% or mean (SD)
**Gender**						
Male	325	34.1%	222	38.1%	166	47.0%
Female	627	65.9%	360	61.9%	187	53.0%
Age (years)	951	49.47 (15.32)	581	54.12 (15.04)	353	55.01 (14.39)
Years of Education	952	16.04 (5.32)	582	15.84 (5.29)	353	14.73 (5.36)
**Household income/week**						
<1,000AUD	216	26.7%	174	36.3%	106	38.1%
1,000–<2,000AUD	234	28.9%	130	27.1%	78	28.1%
≥2,000AUD	360	44.4%	176	36.7%	94	33.8%
**Chronic disease**						
Yes	440	46.2%	287	49.3%	150	42.5%
No	512	53.8%	295	50.7%	203	57.5%
**Weight status**						
Normal	268	28.4%	163	28.3%	69	19.7%
Overweight	312	33.1%	189	32.9%	126	36.0%
Obese	343	36.3%	209	36.3%	150	42.9%
**Depression level**						
Normal	573	60.2%	311	53.4%	227	64.3%
Mild	115	12.1%	73	12.5%	39	11.0%
Moderate	143	15.0%	87	14.9%	41	11.6%
Severe	48	5.0%	35	6.0%	19	5.4%
Extremely severe	73	7.7%	76	13.1%	27	7.6%
**Anxiety level**						
Normal	730	76.7%	425	73.0%	278	78.8%
Mild	55	5.8%	16	2.7%	17	4.8%
Moderate	82	8.6%	63	10.8%	27	7.6%
Severe	27	2.8%	33	5.7%	10	2.8%
Extremely severe	58	6.1%	45	7.7%	21	5.9%
**Stress level**						
Normal	683	71.7%	414	71.1%	273	77.3%
Mild	89	9.3%	46	7.9%	25	7.1%
Moderate	85	8.9%	56	9.6%	26	7.4%
Severe	63	6.6%	36	6.2%	19	5.4%
Extremely severe	32	3.4%	30	5.2%	10	2.8%
**Meeting physical activity guidelines**						
No	404	45.4%	261	47.5%	167	50.9%
Yes	485	54.6%	289	52.5%	161	49.1%
Depression score	952	9.57 (10.17)	582	11.36 (11.44)	353	8.41 (10.36)
Anxiety score	952	4.84 (7.23)	582	5.67 (7.51)	353	4.49 (7.24)
Stress score	952	10.57 (9.76)	582	11.24 (10.29)	353	8.96 (9.83)
Total physical activity minutes/week	889	303.67 (364.71)	550	335.93 (414.27)	328	315.91 (392.45)

### Changes in depression, anxiety, stress, and physical activity

In longitudinal sample, Model 2 shows there was a significant decrease in depression score between time 1 and time 3 (*d* = 1.30, *p* = 0.003, CI = 0.36, 2.24) and between time 2 and time 3 (*d* = 1.03, *p* = 0.009, CI = 0.20, 1.85) (Table 3). There was a significant decrease in anxiety score between time 2 and time 3 (*d* = 0.57, *p* = 0.041, CI = 0.02, 1.12). There was a significant decrease in stress score between time 1 and time 3 (*d* = 1.13, *p* = 0.010, CI = 0.21, 2.04) as well as between time 2 and time 3 (*d* = 0.90, *p* = 0.024, CI = 0.09, 1.70). There were no significant differences in physical activity between the three-time points.

**TABLE 3:** Changes in mental health and physical activity in the longitudinal sample.

	Model 1			Model 2		
	Difference	95% CI	*P*-value	Difference	95% CI	*P*-value
**Depression score**						
Time 1 vs. Time 3	1.28	0.43, 2.13	0.001	1.30	0.36, 2.24	0.003
Time 1 vs. Time 2	0.17	−0.55, 0.89	1.000	0.27	−0.53, 1.07	1.000
Time 2 vs. Time 3	1.11	0.37, 1.85	0.001	1.03	0.20, 1.85	0.009
**Anxiety score**						
Time 1 vs. Time 3	0.29	−0.28, 0.87	0.671	0.31	−0.31, 0.94	0.689
Time 1 vs. Time 2	−0.30	−0.79, 0.19	0.418	−0.25	−0.79, 0.28	0.759
Time 2 vs. Time 3	0.60	0.09, 1.10	0.015	0.57	0.02, 1.12	0.041
**Stress score**						
Time 1 vs. Time 3	1.10	0.27, 1.94	0.005	1.13	0.21, 2.04	0.010
Time 1 vs. Time 2	0.09	−0.62, 0.80	1.000	0.23	−0.55, 1.01	1.000
Time 2 vs. Time 3	1.01	0.28, 1.75	0.003	0.90	0.09, 1.70	0.024
**Total PA minutes**						
Time 1 vs. Time 3	−9.47	−45.10, 26.16	1.000	−9.75	−47.95, 28.45	1.000
Time 1 vs. Time 2	−10.26	−20.14, 40.66	1.000	15.47	−17.17, 48.11	0.768
Time 2 vs. Time 3	−19.73	−51.24, 11.78	0.401	−25.22	−59.16, 8.72	0.225

Model 1 is bivariate. Model 2 accounts for age, gender, educational attainment, income, body mass index, and chronic disease status.

**FIGURE 1:**
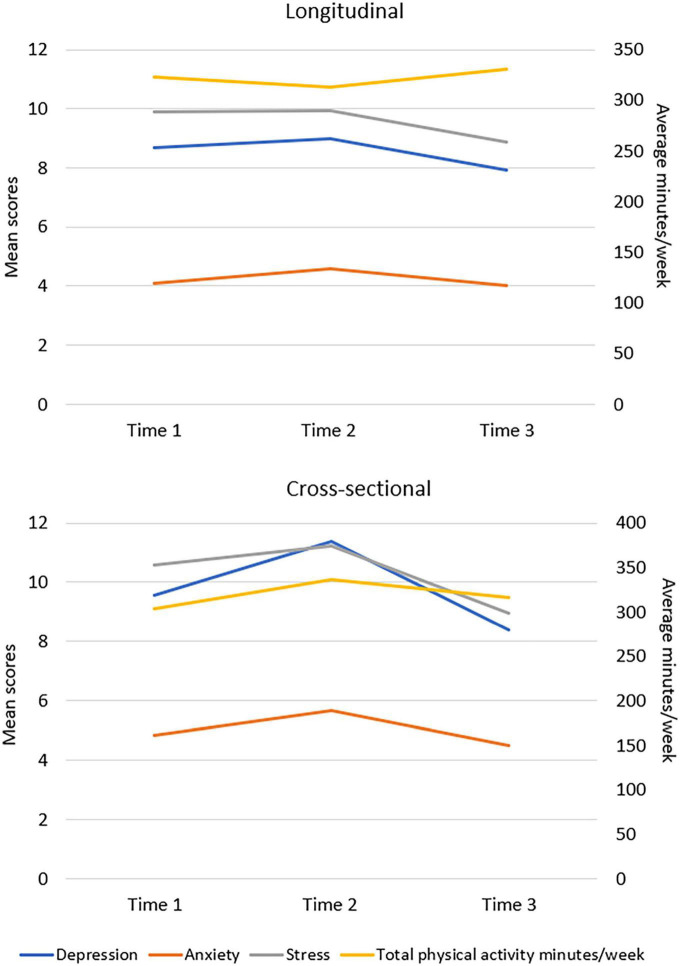
Changes in depression, anxiety, stress, and physical activity over time.

In the cross-sectional sample, Model 2 shows a significant increase in depression score between time 1 and time 2 (*d* = −2.25, *p* = 0.001, CI = −3.72, −0.77) and a significant decrease between time 2 and time 3 (*d* = 2.78, *p* = 0.001, CI = 0.93, 4.64) was found (Table 4). There was also a significant increase in anxiety score between time 1 and time 2 (*d* = −1.03, *p* = 0.032, CI = −2.00, −0.06). There was a significant increase in stress score between time 1 and time 2 (*d* = −1.56, *p* = 0.013, CI = −2.87, −0.25) and significant decrease in stress score between time 2 and time 3 (*d* = 2.18, *p* = 0.006, CI = 0.50, 3.86). There were no significant differences in the physical activity level found between the three-time points. The changes in the outcomes were also visualized in Figure 1.

**TABLE 4:** Changes in mental health and physical activity outcomes in cross-sectional sample.

	Model 1			Model 2		
	Difference	95% CI	*P*-value	Difference	95% CI	*P*-value
**Depression score**						
Time 1 vs. Time 3	1.16	−0.37, 2.70	0.211	0.54	−1.10, 2.18	1.000
Time 1 vs. Time 2	−1.79	−3.17, −0.41	0.006	−2.25	−3.72, −0.77	0.001
Time 2 vs. Time 3	2.95	1.21, 4.69	0.000	2.78	0.93, 4.64	0.001
**Anxiety score**						
Time 1 vs. Time 3	0.35	−0.73, 1.43	1.000	0.02	−1.15, 1.20	1.000
Time 1 vs. Time 2	−0.83	−1.76, 0.11	0.102	−1.03	−2.00, −0.06	0.032
Time 2 vs. Time 3	1.18	−0.01, 2.36	0.052	1.05	−0.22, 2.33	0.142
**Stress score**						
Time 1 vs. Time 3	1.61	0.14, 3.07	0.026	0.62	−0.89, 2.13	0.980
Time 1 vs. Time 2	−0.67	−1.94, 0.60	0.611	−1.56	−2.87, −0.25	0.013
Time 2 vs. Time 3	2.28	0.67, 3.90	0.002	2.18	0.50, 3.86	0.006
**Total PA minutes**						
Time 1 vs. Time 3	−12.24	−71.73, 47.25	1.000	12.65	−48.57, 73.87	1.000
Time 1 vs. Time 2	−32.25	−83.65, 19.14	0.399	−14.43	−68.25, 39.39	1.000
Time 2 vs. Time 3	20.01	−46.83, 86.86	1.000	27.08	−38.46, 92.63	0.968

Model 1 is bivariate. Model 2 accounts for age, gender, educational attainment, income, body mass index, and chronic disease status.

### Associations between mental health and physical activity

Table 5 shows there were significant associations between depression, anxiety, and stress with physical activity in both longitudinal and cross-sectional samples. Particularly, Model 2 shows that compared to those who reported not meeting the physical activity guidelines, those who reported meeting the physical activity guidelines were found to have significantly lower depression (*d* = −2.08, *p* = 0.001, CI = −2.90, −1.26), anxiety (*d* = −0.88, *p* = 0.001, CI = −1.41, −0.34) and stress scores (*d* = −1.35, *p* = 0.001, CI = −2.13, −0.56) in the longitudinal sample, and also significant lower depression (*d* = −2.32, *p* = 0.001, CI = −3.41, −1.23), anxiety (*d* = −1.45, *p* = 0.001, CI = −2.21, −0.70) and stress (*d* = −1.44, *p* = 0.005, CI = −2.44, −0.44) scores in the cross-sectional sample.

**TABLE 5:** Associations between mental health scores and meeting physical activity guidelines.

	Model 1			Model 2		
	Difference	95% CI	*P*-values	Difference	95% CI	*P*-values
**Longitudinal sample**						
Depression score	−2.03	−2.78, −1.28	<0.001	−2.08	−2.90, −1.26	<0.001
Anxiety score	−0.93	−1.43, −0.42	<0.001	−0.88	−1.41, −0.34	0.001
Stress score	−1.19	−1.92, −0.46	0.002	−1.35	−2.13, −0.56	<0.001
**Cross-sectional sample**						
Depression score	−2.82	−3.81, −1.84	<0.001	−2.32	−3.41, −1.23	<0.001
Anxiety score	−1.94	−2.61, −1.26	<0.001	−1.45	−2.21, −0.70	<0.001
Stress score	−1.76	−2.69, −0.83	<0.001	−1.44	−2.44, −0.44	0.005

Model 1 includes meeting physical activity (yes vs. no) and time. Model 2 includes meeting physical activity (yes vs. no), time, age, gender, educational attainment, income, body mass index, and chronic disease status.

## Discussion

The present study examined changes in depression, anxiety, stress and physical activity, as well as associations between depression, anxiety and stress with physical activity in Australian adults across three-time points during the COVID-19 pandemic. The findings showed that depression, anxiety, and stress scores were generally lower at time 3 for both longitudinal and cross-sectional samples. There was, however, one exception to this, the cross-sectional data indicated that from time 1 to time 2, depression, anxiety and stress scores significantly increased and the proportion of participants reporting symptoms of extremely severe depression and severity anxiety, were doubled. This may in part be due to the negative impact of two consecutive lockdowns in Victoria, as there was an increase in the number of participants from Victoria joining the cross-sectional sample from 16.7 to 36.6% at time 2. The negative impact of strict lockdown measures on mental health have been reported in previous studies (Brooks et al., 2020; Chandola et al., 2020; Rossi et al., 2020). Similarly, our results shows that depression, anxiety and stress improved as the lockdown measures were relaxed.

Our findings are also in line with the cross-sectional research outcomes reported by the Australian Bureau of Statistics, where in December 2020, the number of Australian adults feeling negatively impacted by the COVID-19 pandemic in relation to mental health, reduced to its lowest level since the initial peak in April 2020 (Australian Institute of Health and Welfare, 2021a). Our findings, however, are in contrast with reviews suggesting a worsening of mental health during the COVID-19 pandemic (Salari et al., 2020; Violant-Holz et al., 2020; Xiong et al., 2020). These differences may be accounted for by a combination of factors related to differences in both pandemic management and study sample. Since the onset of the COVID-19 pandemic, the Australian government has significantly invested in mental health care and unprecedented fiscal measures to support the nation’s wellbeing and psychosocial functioning (Australian Institute of Health and Welfare, 2021b; Chen and Langwasser, 2021). Furthermore, when compared to most countries being affected by the COVID-19 pandemic, Australia had lower infection and mortality rates, and implemented less rounds of lockdown, which helped to minimize disruptions to usual living conditions to the greatest extent possible (Lowly Institute, 2020; Australian Institute of Health and Welfare, 2021a). In addition, a large proportion of our participants reported having a higher household income, being older and having more years of education than the Australian general population. Previous studies suggest these sociodemographic factors may be protective for mental health and wellbeing during the COVID-19 pandemic (Coulombe et al., 2020; Newby et al., 2020; Kunzler et al., 2021).

In contrast with studies reporting significant reductions in physical activity during the COVID-19 pandemic (Violant-Holz et al., 2020; Faulkner et al., 2021; Karageorghis et al., 2021; Stockwell et al., 2021), our findings indicated no significant changes in physical activity for both longitudinal and cross-sectional samples across the three-time points. The total average weekly physical activity reported by our participants was also marginally higher than the pre-pandemic general population average of 294 min of weekly physical activity for Australians aged 15 and over (Australian Bureau of Statistics, 2018). More than 55% of the longitudinal sample and 49% of the repeated cross-sectional sample reported meeting the physical activity guidelines of accumulating at least 150 min of moderate intensity physical activity per week, which were slightly higher than the pre-pandemic Australian general population average of 45% of adults meeting the physical activity guidelines (Vandelanotte et al., 2010; Alley et al., 2017; Australian Bureau of Statistics, 2018). Nevertheless, this is still a concerning low level of physical activity considering the significant health risks and related economic cost associated with physical inactivity (Ding et al., 2016; Hall et al., 2021).

The consistent levels of physical activity found in our samples may be due to several protective factors. Australian governments have actively promoted benefits and practical access to physical activity during the pandemic, including permitting outdoor exercise and recreational activities to be essential reasons for leaving home even during lockdown periods (Fitzpatrick et al., 2020). This was also reflected in the uptake and engagement of the 10,000 Steps program which quadrupled during the pandemic, with thousands of new people registering with the program (To et al., 2021). The lower infection rate and effective containment of the COVID-19 have also provided the public with a safer outdoor environment to be physically active (Lowly Institute, 2020; Australian Institute of Health and Welfare, 2021a). In addition, previous research has identified people being older in age, mentally well and having a higher household income and education level, tended to be more physically active during the COVID-19 pandemic, and most of our participants also shared these sociodemographic characteristics (Bu et al., 2021).

Consistent with previous evidence, the present study also demonstrated that regular participation in physical activity is associated with lower scores for depression, anxiety, and stress (Stubbs et al., 2017; Chekroud et al., 2018; Callow et al., 2020; Gianfredi et al., 2020; Lesser and Nienhuis, 2020; Stanton et al., 2020; Puccinelli et al., 2021). The positive influence of physical activity on mental health holds significant importance and implications for people’s wellbeing and health preventative measures during the COVID-19 pandemic. Governments and health service providers should continue to promote the benefits and access to physical activity during this unprecedented health crisis and beyond. In particular, US and many European countries have already taken the initiative to invest and reconfigure streets into walking and cycling infrastructures during the pandemic to promote physical activity within a dedicated space (Doubleday et al., 2021; Kraus and Koch, 2021).

The present study has several strengths, including large sample sizes, broad national inclusion of participants from metropolitan, regional, and remote areas of Australia and application of both longitudinal and cross-sectional study designs. However, it also has limitations, which should be considered when interpreting and generalizing the results to other populations. Firstly, this study does not include pre-pandemic data and it is therefore unknown how mental health and physical activity outcomes changed from before the pandemic to the first time point. Secondly, a large proportion of participants were female, older in age, had higher household incomes, and more years of education than the Australian adult population. Thirdly, this study also relied on voluntary participation and there may have been an over-representation of participants joining in the study who perceived themselves to be affected by the current pandemic. Fourthly, this is an observational study and therefore, it is not possible to control for unknown confounders. Finally, the use of self-reported measures could result in recall bias and further research should consider using objective measures to strengthen the findings.

In conclusion, our findings show an improvement in depression, anxiety, and stress and no significant changes in physical activity in Australian adults across three-time points during the COVID-19 pandemic. The significant benefits of engaging in sufficient physical activity on mental health were also demonstrated. These findings have practical implications for physical activity to be utilized as a cost-effective coping method to support people’s mental health, and the need for governments and service providers to continue implement initiatives to promote mental wellbeing and physical activity as the COVID-19 pandemic continues to evolve. The findings from this study may also be applied in similar contexts in which other extraordinary negative events cause large scale psychological distress and changed social conditions (e.g., war, new pandemic). Further research is needed to monitor the long-term impact of the COVID-19 pandemic on mental health and physical activity to inform and guide appropriate public health promotion and intervention in a timely manner.

## Data availability statement

The raw data supporting the conclusions of this article will be made available by the authors, without undue reservation.

## Ethics statement

The studies involving human participants were reviewed and approved by the Human Research Ethics Committee of Central Queensland University, Australia (Approval number: 22332). The patients/participants provided their written informed consent to participate in this study.

## Author contributions

QT, RS, SK, SW, SA, TT, AF, and CV: conceptualization, methodology, and review and editing. TT: data curation. WZ and QT: data analysis. WZ: manuscript drafting. All authors contributed to the article and approved the submitted version.
